# Hepatic steatosis is associated with abnormal hepatic enzymes, visceral adiposity, altered myocardial glucose uptake measured by ^18^F-FDG PET/CT

**DOI:** 10.1186/s12902-020-00556-x

**Published:** 2020-05-27

**Authors:** Lijun Hu, Xiaoliang Shao, Chun Qiu, Xiaonan Shao, Xiaosong Wang, Rong Niu, Yuetao Wang

**Affiliations:** 1 Department of Radiation Oncology, The Affiliated Changzhou No. 2 People’s Hospital of Nanjing Medical University, Changzhou, 213003 Jiangsu China; 2 Department of Nuclear Medicine, The Third Affiliated Hospital of Soochow University, Changzhou, 213003 Jiangsu China

**Keywords:** Nonalcoholic fatty liver disease, ^18^F-Fluorodeoxyglucose, Positron emission tomography, Myocardial, Visceral adipose tissue

## Abstract

**Background:**

Nonalcoholic fatty liver disease (NAFLD) is a multisystem disease that affects the liver and a variety of extra-hepatic organ systems. This study aimed to investigate the relationship between hepatic steatosis and glucose metabolism in liver and extra-hepatic tissues and organs.

**Methods:**

The whole body ^18^F-fluorodeoxyglucose (FDG) positron emission tomography (PET)/computed tomography (CT) images of 191 asymptomatic tumor screening patients were retrospectively analyzed. Patients with the ratio of spleen/liver CT densities > 1.1 were defined to have NAFLD, and their clinical symptoms, laboratory markers, FDG uptake in a variety of tissues and organs including heart, mediastinal blood pool, liver, spleen, pancreas, and skeletal muscle, as well as abdominal adipose tissue volumes including visceral adipose tissue (VAT) volume and subcutaneous adipose tissue (SAT) volume were compared with those of the non-NAFLD patients and used to analyze the independent correlation factors of NAFLD.

**Results:**

Among the 191 patients, 33 (17.3%) were NAFLD, and 158 (82.7%) were non-NAFLD. There was no significant correlation between the mean standardized uptake value (SUVmean) and CT density of liver as well as the ratio of spleen/liver CT densities. Hepatic steatosis, but not FDG intake, was more significant in NAFLD patients with abnormal liver function than those with normal liver function. Compared with the non-NAFLD patients, NAFLD patients had significantly reduced myocardial glucose metabolism, but significantly increased mediastinal blood pool, spleen SUVmean and abdominal adipose tissue volumes (including VAT and SAT volumes) (*P* < 0.05). Multivariate regression analysis showed that elevated serum ALT, increased abdominal VAT volume, and decreased myocardial FDG uptake were independent correlation factors for NAFLD. Further studies showed that hepatic steatosis and myocardial FDG uptake were mildly linearly correlated (*r* = 0.366 with hepatic CT density and − 0.236 with the ratio of spleen/liver CT densities, *P* < 0.05).

**Conclusions:**

NAFLD is a systemic disease that can lead to the change of glucose metabolism in some extra-hepatic tissues and organs, especially the myocardium.

## Background

Nonalcoholic fatty liver disease (NAFLD) is the most common liver disease worldwide, with a prevalence exceeding 15% in China [1]. It is an acquired metabolic stress-related liver disorder due to the comprehensive effects of multiple factors such as abnormalities in glucose and lipid metabolism, insulin resistance, and inflammation. In the majority of patients, NAFLD is commonly associated with metabolic comorbidities such as obesity, diabetes mellitus, and dyslipidemia [2]. Studies have shown that NAFLD patients, especially those with nonalcoholic steatohepatitis (NASH), are at increased risk of mortality from liver diseases (13%), and more commonly from cardiovascular diseases (25%) and malignancies (28%) [3]. Early identification of clinical risk factors (such as biochemical indicators, imaging changes, etc.) by monitoring disease progression will help early identification of high-risk patients (NASH, cardiovascular diseases) and timely intervention. ^18^F-fluorodeoxyglucose (FDG) positron emission tomography (PET)/computed tomography (CT) can reflect fasting glucose metabolism in a variety of tissues and organs and is an ideal tool theoretically for screening high-risk patients for NAFLD. Thus, it is widely used in tumor, cardiovascular, and neurological fields. However, for complex metabolic disorders, it is inconclusive whether abnormalities in a variety of tissues and organs, especially in extra-hepatic glucose metabolism, are related to the progression or complications of NAFLD. Therefore, the present study aimed to investigate the association between hepatic steatosis and glucose metabolic abnormality in a variety of organs/tissues assessed by using ^18^F-FDG PET/CT in a population-based cohort.

## Methods

### Research subjects

Patients who admitted to the Physical Examination Center of our hospital from October 2010 to December 2015 and were subjected to whole-body ^18^F-FDG PET/CT imaging due to a variety of reasons (unexplained tumor indicators, tumor family genetic history, suspicious carcinogen exposure history or tumor screening) were retrospectively analyzed. Patients were excluded if they had alcohol consumption ≥20 g/day (for women) or ≥ 30 g/day (for men) [4], acute/chronic hepatitis, history of coronary heart diseases, myocardial infarction or cardiomyopathy, history of malignancy, vasculitis or collagen diseases, history of use of drugs that could cause hepatic steatosis (such as sodium valproate, methotrexate, etc.). Patients’ information, including gender, age, height, weight, and smoking status (excluding passive smoking) were recorded. Patients with body weight index (BMI) of 24–28 kg/m^2^ were considered as overweight as defined by the diagnostic criteria of the Chinese Obesity Working Group [5], and patients with BMI ≥ 28 kg/m^2^ were diagnosed as obesity. All patients signed the informed consent form. The protocol of this study adhered to the Declaration of Helsinki and was approved by the Ethics Committee of our hospital.

### Measurements and definitions of laboratory parameters

A total of 5 ml of venous blood sample was collected from each patient who had been fastened for at least 8 h before ^18^F-FDG PET/CT scanning and used for determination of 1) alanine aminotransferase (ALT), aspartate aminotransferase (AST), total cholesterol (TC), triglyceride (TG), high-density lipoprotein cholesterol (HDL-C), low-density lipoprotein cholesterol (LDL-C) and fasting blood glucose using Hitachi 7600–120 automatic biochemical analyzer, 2) thyroid stimulating hormone (TSH) and fasting insulin levels using Roche Cobas 8000 automatic electrochemical immunoassay analyzer, and 3) fasting glycosylated hemoglobin (HbA1c) using high-pressure liquid phase method on Bio-Rad D-10™ Hemoglobin Analyzer. Patients with ALT ≥50 u/L were considered to have abnormal liver function. Patients who had been diagnosed as diabetes or who had fasting blood glucose ≥7.0 mmol/L or HbA1c ≥ 6.5% were diagnosed as diabetes according to the 2017 Standards of Medical Care in Diabetes issued by American Diabetes Association [6]. According to the guidelines [7], patients who met at least one of the following criteria: serum TC ≥ 6.22 mmol/L, serum TG ≥ 2.26 mmol/L, serum HDL-C < 1.04 mmol/L and serum LDL-C ≥ 4.14 mmol/L were diagnosed as dyslipidemia. The homeostasis model assessment of insulin resistance (HOMA-IR) index was calculated according to the following formula [8]: fasting insulin (mIU/L) × fasting glucose (mg/dL)/405. HOMA-IR > 2.5 was defined as insulin resistance [9].

### PET/CT scan

PET scan was performed using ^18^F-FDG with radiation purity > 95% as the developer on a German Siemens Biograph mCT (64) PET/CT instrument. Before intravenous injection of ^18^F-FDG at 3.70–5.18 MBq/kg, all subjects fasted for > 8 h and their fasting blood glucose were determined. PET/CT scan was performed at 45–60 min after injection of ^18^F-FDG. CT scan was performed using CareDose 4D technology at tube voltage of 100 kV, pitch of 0.8, bulb single layer rotation time of 0.5 s, layer thickness of 5 mm, and reference of 60–180 mAs. 3D PET scan was conducted by scanning from skull base to upper femur with acquisition of 2 min per bed. The cross-sectional, coronal, sagittal tomographic images, and three-dimensional projection images were reconstructed using the Syngo TureD system [10].

### Definition of NAFLD and determination of glucose metabolism

NAFLD was diagnosed based on the CT images. In brief, four regions of interest (ROI) with a diameter of 4 cm were delineated in the left hepatic lobe, the right hepatic anterior lobe, as well as the upper and lower right hepatic lobes [11], respectively, as well as 2 ROI with diameters of 2 cm were delineated in the spleen with depth intervals of 1.5 cm [12]. Care was taken to avoid biliary structures, major arterial and venous vessels, and the area of the hepatic cyst. The standard uptake value (SUV) was calculated as regional radioactivity concentration (Bq/ml)/injected ^18^F-FDG activity (Bq)/body mass (g). The average CT value and mean SUV (SUVmean) of the liver and spleen were calculated. Patients with the ratio of average CT values of spleen/liver > 1.1 were diagnosed as NAFLD [13]. The liver and spleen SUVmean indicated the level of glucose metabolism in the liver and spleen.

### Determination of glucose metabolism levels in other organs

The left ventricular myocardial ROI was delineated layer by layer on the tomographic PET/CT fusion images. The SUVmean of ^18^F-FDG in the entire left ventricular myocardium was automatically calculated by Siemens TrueD software. Because the glucose uptake of the myocardium was affected by a variety of metabolic substrates, the value varied greatly. For subjects with low myocardial uptake as a blood pool, an ROI with double U-shaped lines with about 1 cm distance was delineated along the border of the left ventricle on the transaxial PET/CT images [11, 14, 15]. The ROI of the mediastinal blood pool was delineated on the continuous layers of the ascending aorta. In the pancreas, three ROIs with a diameter of 1.0 cm were delineated in the pancreatic head, body and tail and used to calculate the SUVmean of the pancreas. The ROI of skeletal muscle was delineated on the upper part of the bilateral femur [16].

### Calculation of abdominal fat volume

The visceral adipose tissue (VAT) volume and subcutaneous adipose tissue (SAT) volume were calculated using CT volume quantification software. The anterior vertebral body was delineated from the first sacrum up to the abdominal wall muscles and the VAT and SAT volumes were divided into 25 consecutive layers with a total length of 125 mm [17]. Fat was defined as any voxel between − 190 to − 60 HU, and the volumes of VAT and SAT were automatically calculated and recorded.

### Statistical analysis

Statistical analysis was performed using SPSS 23.0 statistical software. The measurement data was first tested by Kolmogorov-Smirnov for normal distribution. Those with normal distribution were expressed as mean ± standard deviation. The mean values of two groups were compared using t test for two independent samples. The mean values of multiple groups were compared using one-way ANOVA. Data without normal distribution were expressed as the median (P25, P75), and the medians of two groups were compared using the Mann-Whitney U test. The count data were expressed as the ratio or percentage and compared using the χ^2^ test. Correlation between continuous variables in univariate analysis was analyzed using Pearson correlation analysis, and the correlation between grading variables was analyzed using Spearman correlation analysis. The independent correlation factors for NAFLD were analyzed using logistic multivariate regression analysis of two categorical variables, and the regression coefficient, odds ratio (OR) and 95% confidence interval were calculated. A *P* < 0.05 measured by two-sided test was considered statistically significant [11].

## Results

### Characteristics of the subjects

A total of 191 patients were enrolled in the study. They were 48.8 ± 9.0 years old on average and had mean BMI of 24.8 ± 2.9 kg/m^2^. Among them, males accounted for 67.5% (129/191); overweight and obesity patients accounted for 60.7% (116/191); smokers accounted for 40.8% (78/191); diabetic patients accounted for 13.1% (25/191); and insulin resistant patients accounted for 18.8% (36/191). Table 1 lists the clinical characteristics and laboratory indicators of the studied population.

**Table 1:** Demographic, laboratory, and clinical characteristics of the enrolled subjects

Parameters	Overall sample (*n* = 191)	NAFLD (*n* = 33)	Non-NAFLD (*n* = 158)	*P* value
Male (%)	67.5	81.8	64.6	0.054
Age (years)	48.8 ± 9.0	47.2 ± 7.4	49.1 ± 9.3	0.290
BMI (kg/m^2^)	24.8 ± 2.9	27.0 ± 2.8	24.4 ± 2.7	< 0.001^*^
Current smokers (%)	40.8	54.5	38.0	0.078
Diabetes mellitus (%)	13.1	27.3	10.1	0.008^*^
Fasting glucose (mmol/L)	5.83 ± 1.19	6.28 ± 1.33	5.74 ± 1.15	0.017^*^
Insulin resistance (%)	18.8	45.5	13.3	< 0.001^*^
HbA1c (%)	5.40 (5.20, 5.80)	5.50 (5.30, 6.10)	5.40 (5.20, 5.70)	0.043^*#^
Fasting insulin (mIU/L)	6.01 (4.63, 8.52)	9.44 (7.71, 10.46)	5.54 (4.46, 7.50)	< 0.001^*#^
HOMA-IR	1.53 (1.11, 2.31)	2.48 (2.07, 2.88)	1.41 (1.05, 1.98)	< 0.001^*#^
Dyslipidemia (%)	64.9	93.9	58.9	< 0.001^*^
TC (mmol/ L)	5.05 ± 1.04	5.41 ± 0.99	4.98 ± 1.03	0.029^*^
TG (mmol/ L)	2.29 (1.63, 3.21)	3.19 (2.55, 4.88)	2.15 (1.53, 2.84)	0.001^*#^
HDL-C (mmol/ L)	1.17 ± 0.28	0.98 ± 0.18	1.21 ± 0.28	< 0.001^*^
LDL-C (mmol/ L)	2.50 ± 0.64	2.67 ± 0.67	2.46 ± 0.64	0.085
TSH (μIU/ml)	2.09 (1.51, 3.09)	2.70 (1.79, 3.50)	2.05 (1.47, 2.95)	0.035^*#^
ALT (u/L)	25.0 (18.0, 34.0)	35.0 (29.0, 47.5)	22.0 (17.0, 30.0)	< 0.001^*#^
AST (u/L)	19.3 ± 5.6	25.6 ± 6.4	18.6 ± 5.2	< 0.001^*^

*NAFLD* Nonalcoholic fatty liver disease, *BMI* Body mass index, *TC* Total cholesterol, *TG* Triglyceride, *HDL-C* High density lipoprotein cholesterol, *LDL-C* Low density lipoprotein cholesterol, *TSH* Thyroid stimulating hormone, *ALT* Alanine aminotransferase, *AST* Aspartate aminotransferase. HOMA-IR = fasting glucose × fasting insulin / 22.5

* indicates significant statistical differences, # indicates using Mann-Whitney U-test

### Relationship between hepatic steatosis and hepatic ^18^F-FDG uptake

**Fig. 1:**
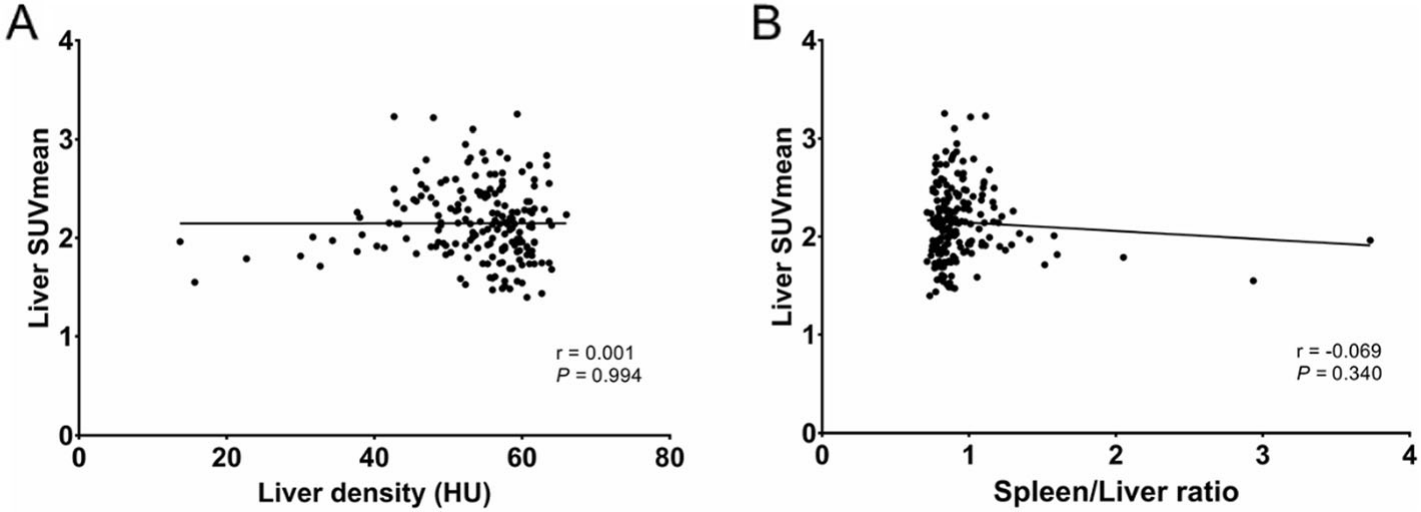
Relationship between hepatic steatosis and hepatic ^18^F-FDG uptake. There was no significant correlation between hepatic steatosis and glucose metabolism (*P* > 0.05)

Among the 191 enrolled patients, 33 were NAFLD, accounting for 17.3%, and 158 were non-NAFLD, accounting for 82.7%. The liver CT density was 40.5 ± 9.3 HU in average, and the ratio of spleen/liver CT densities was 1.36 ± 0.56 in NAFLD patients, showing the significant difference from that of 56.8 ± 4.2 HU and 0.86 ± 0.08 in non-NAFLD patients (*P* < 0.001), respectively. The liver SUVmean was 2.14 ± 0.35 in NAFLD patients, showing no significant difference from that of 2.15 ± 0.35 in non-NAFLD patients (*P* = 0.856). Liver SUVmean was not significantly correlated with liver CT density, as well as the ratio of spleen/liver CT densities before (*r* = 0.001 and − 0.069, *P* = 0.994 and 0.340, Fig. 1) and after adjusting to ^18^F-FDG injection (*r* = 0.013 and − 0.083, *P* = 0.859 and 0.255), as shown in Fig. 1.

**Fig. 2:**
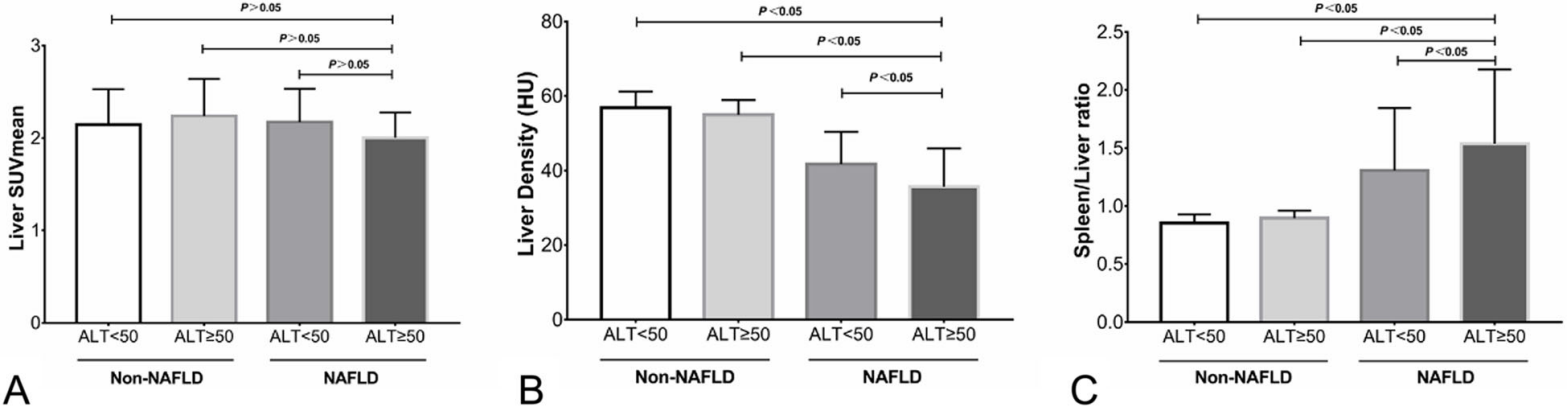
Comparison of hepatic ^18^F-FDG uptake (**a**), CT density (**b**) and the ratio of spleen/liver CT densities (**c**) between non-NAFLD patients with normal liver function and non-NAFLD patients with abnormal liver function. Patients with ALT≥50 u/L was considered to have abnormal liver function

Among the 33 NAFLD patients, patients with abnormal liver function accounted for 21.2% (7/33). Their liver CT density was 35.7 ± 10.3 HU, and the ratio of spleen/liver CT densities was 1.54 ± 0.64, which were significantly different from that of 41.7 ± 8.7 HU and 1.31 ± 0.54 in patients with normal liver function (*P* < 0.05). Their liver SUVmean was 2.00 ± 0.27, which was not significantly different from that of 2.17 ± 0.35 in patients with normal liver function (*P* > 0.05), as shown in Fig. 2.

### Comparison of PET/CT images between NAFLD and non-NAFLD patients

**Fig. 3:**
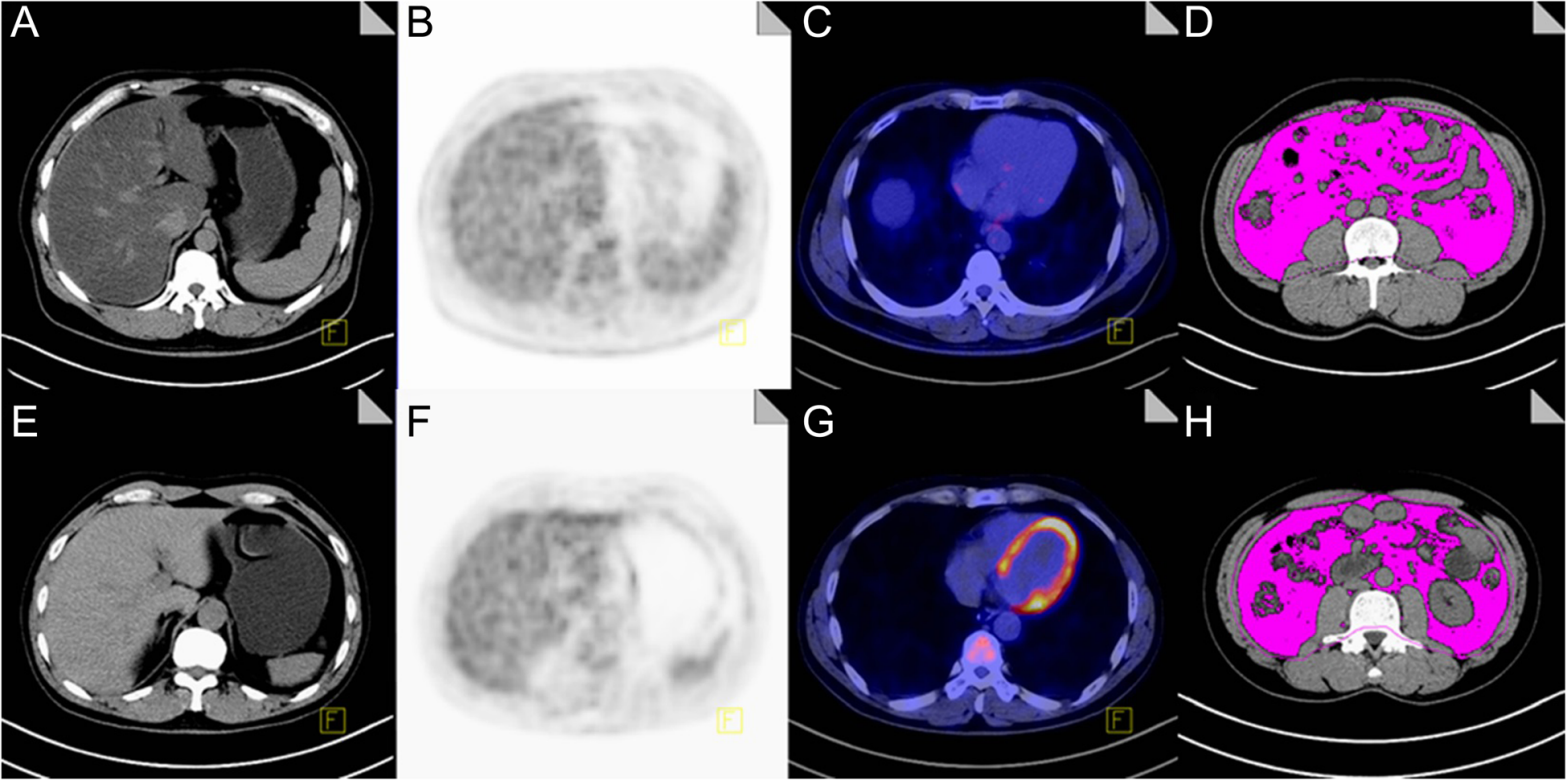
Comparison of PET/CT images between NAFLD patients and non-NAFLD patients. **a**-**d** are the images of a 45-year-old male, showing **a** significantly reduced liver CT density, liver CT value of 15.67 HU, and the ratio of spleen/liver CT intensities of 2.94; **b** liver SUVmean of 1.55; **c** no significant FDG uptake in the myocardium; **d** VAT volume of 2250 cm^3^. **e**-**h** are the images of a 50-year-old male, showing **e** liver CT value of 56.00 HU, and the ratio of spleen/liver CT intensities of 0.74; **f** liver SUVmean of 1.81; **g** significant FDG uptake in myocardium; **h** VAT volume of 1552 cm^3^

Compared with the non-NAFLD patients, patients with NAFLD had significantly decreased myocardial glucose metabolism (*P* < 0.05), slightly but significantly elevated mediastinal blood pool and spleen SUVmean (*P* < 0.05), significantly increased abdominal fat volume (including VAT and SAT volumes) (*P* < 0.05), and similar pancreatic and skeletal muscle SUVmean (*P* < 0.05), as shown in Fig. 3. Table 2 shows the SUVmean of body PET/CT and abdominal fat volume.

**Table 2:** Comparison of PET/CT images between NAFLD patients and non-NAFLD patients

Parameters	NAFLD (*n* = 33)	Non-NAFLD (*n* = 158)	*P* value
Myocardial SUVmean	1.46 (1.19, 2.73)	3.06 (1.67, 4.57)	<0.001^*#^
Mediastinal blood pool SUVmean	1.49 ± 0.31	1.34 ± 0.33	0.015^*^
Liver SUVmean	2.14 ± 0.35	2.15 ± 0.35	0.856
Spleen SUVmean	1.73 ± 0.19	1.61 ± 0.22	0.005^*^
Pancreas SUVmean	1.45 ± 0.18	1.49 ± 0.26	0.487
Skeletal muscle SUVmean	0.68 ± 0.14	0.66 ± 0.14	0.603
Abdominal adipose volume (cm^3^)	4040 (3096, 4552)	3043 (2480, 3617)	<0.001^*#^
VAT volume (cm^3^)	1857 (1516, 2118)	1230 (882, 1652)	<0.001^*#^
SAT volume (cm^3^)	2042 (1517, 2570)	1725 (1432, 2124)	0.017^*#^

*VAT* Visceral adipose tissue, *SAT* Subcutaneous adipose tissue

* indicates significant statistical differences, # indicates using Mann-Whitney U-test

### Analysis of the factors related to NAFLD

**Fig. 4:**
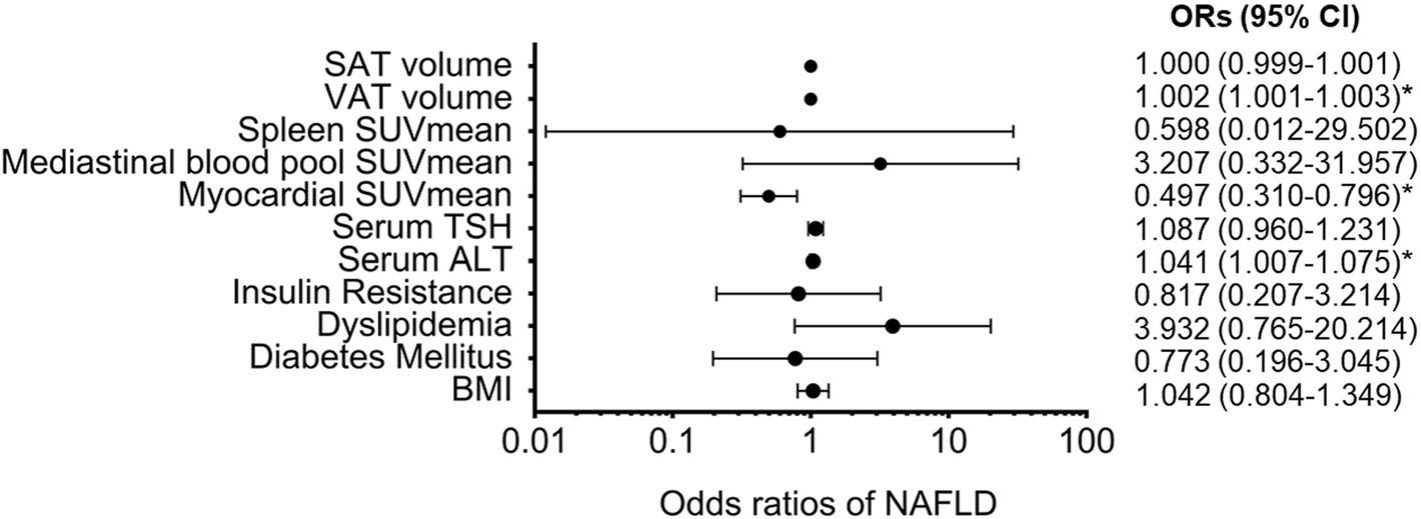
Analysis of factors related to NAFLD. * indicates statistically significant differences

Univariate analysis of the relationship between NAFLD and clinical features, blood biochemical parameters, glucose metabolism levels of different tissues, as well as abdominal fat volume and distribution showed that NAFLD had significant positive correlations with BMI, diabetes, insulin resistance, serum TSH, ALT and AST, mediastinal blood pool, spleen SUVmean, VAT and SAT volumes, with the correlation coefficient *r* = 0.154–0.390 (*P* < 0.05), but significant negative correlation with myocardial SUVmean with correlation coefficient *r* = − 0.320 (*P* < 0.001). Logistic regression analyses of these factors showed that elevated serum ALT, increased VAT volume, and reduced myocardial SUVmean were independent risk factors for NAFLD (*P* < 0.05). Figure 4 shows their OR values and 95%CI.

**Fig. 5:**
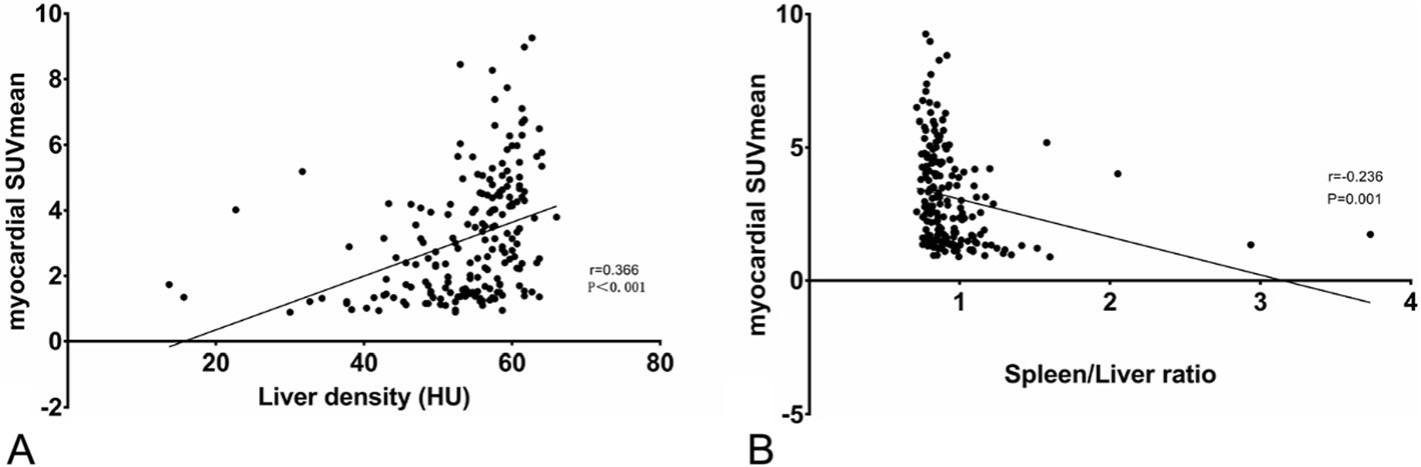
Correlation between hepatic steatosis and myocardial glucose uptake

Further analyses of the correlation between hepatic steatosis (hepatic CT density and the ratio of spleen/liver CT densities) and myocardial glucose uptake (SUVmean) found that myocardial SUVmean had a significant positive correlation with hepatic CT density (*r* = 0.366, *P* < 0.001) and a significant negative correlation with the ratio of spleen/liver CT densities (*r* = − 0.236, *P* = 0.001), as shown in Fig. 5.

## Discussion

Our study indicated that NAFLD patients are usually accompanied by obesity, diabetes, insulin resistance, dyslipidemia and hypothyroidism, etc., which is now regarded as a comprehensive hepatic manifestation of metabolic syndrome [2]. By definition, hepatic steatosis is the component and hallmark of NAFLD. We found that in NAFLD patients, hepatic steatosis is independently associated with elevated hepatic enzymes, increased VAT volume, and decreased myocardial FDG uptake, but not with hepatic FDG uptake.

^18^F-FDG is an emerging widely used radiopharmaceutical agent for imaging inflammation because activated inflammatory cells display increased FDG accumulation. Some studies [18, 19] have found that hepatic ^18^F-FDG uptake is significantly higher in NAFLD patients than the controls, suggesting that the development mechanism of NAFLD may be related to non-specific uptake of ^18^F-FDG by NASH inflammatory cells. However, other studies [20, 21] found that there was no significant correlation between NAFLD and ^18^F-FDG uptake, which is consistent with our findings that there was no significant difference in hepatic ^18^F-FDG uptake between NAFLD patients with abnormal liver function (ALT ≥50 u/L) and NAFLD patients with normal liver function (*P* > 0.05). The theoretical basis behind this finding may be 1) a potential ‘dilutional’ effect of hepatic fat on the FDG signal [18]; 2) most NAFLD patients are accompanied with insulin resistance, which could lead to decreased hepatic glycogen synthesis and enhanced glycogen decomposition; and 3) NASH not only expresses as inflammatory cell infiltration but also is accompanied by hepatocyte injury and collagen fiber deposition [22], resulting in a decrease in ^18^F-FDG uptake, which compensate the effect of non-specific uptake of ^18^F-FDG by hepatic inflammatory cells.

Over the past 10 years, it has also become increasingly clear that NAFLD is a multisystem disease that affects a variety of extra-hepatic organs [23]. In this study, we for the first time utilized PET/CT to observe the differences in glucose metabolism of different tissues/organs between NAFLD patients and non-NAFLD subjects at fasting state and found that NAFLD patients had decreased myocardial glucose metabolism and slightly increased mediastinal blood pool and spleen SUVmean. In addition, we also found that decreased myocardial glucose metabolism was a risk factor for NAFLD (OR = 0.497, *P* < 0.05) independent of BMI, diabetes, dyslipidemia, and insulin resistance, consistent with previous research results [11, 21–23]. But the mechanism remains unknown. Some studies showed that individuals with NAFLD and decreased myocardial glucose uptake on FDG PET had higher risk of left ventricular diastolic dysfunction [21, 24, 25]. Tang et al. reported that the low myocardial SUV was independently associated with NAFLD, non-calcified plaque, and significant coronary stenosis [26].

We thought that the existence of systemic insulin resistance in NAFLD patients was the potential mechanism of decreased myocardial glucose metabolism. In this study, a slight but significant increase in blood pool SUVmean in NAFLD patients is indirect evidence of peripheral tissue insulin resistance and glucose metabolism utilization disorders. At the insulin resistance state, the serum concentration of free fatty acids (FFAs) elevated [3], which was another myocardial energy substrate. The elevated FFAs can inhibit pyruvate dehydrogenase (PDH) and glucokinase, which then led to the inhibition of myocardial glucose oxygenation [27]. Abnormal myocardial energy substrate utilization and altered metabolic pathways will lead to “metabolic remodeling” and subsequent cardiac structure and dysfunction [21]. Perseghin et al. [28] found in their study using cardiac ^31^P-MR spectroscopy that NAFLD patients had reduced myocardial phosphocreatine/adenosine triphosphate ratio, suggesting that NAFLD patients have myocardial energy metabolism disorder. Our study further verified this view from the perspective of myocardial energy substrate utilization and found that there is a mild linear correlation between hepatic steatosis and FDG uptake in the myocardium. We thought that this might be an early manifestation of cardiac metabolic remodeling in patients with NAFLD, and cardiac metabolic remodeling may take place precede the development of functional and structural remodeling of the heart [29]. The current evidence from the published prospective studies supports that NAFLD, irrespective of its diagnostic methodology, was significantly associated with an increased risk of fatal and nonfatal cardiovascular events [30]. But direct evidence of the correlation between myocardial glucose reduction and cardiovascular risk remains to be confirmed.

Our study once again confirmed that VAT volume and serum ALT are independently associated with NAFLD. Although the median ALT value of NAFLD patients was within the normal range, the degree of hepatic fat infiltration was significantly higher in patients with ALT ≥50 u/L than in patients with ALT < 50 u/L (*P* < 0.05). As we know, ALT is a serum marker for liver inflammation or injury and frequently elevated in NAFLD patients. But persistently elevated ALT levels may be the risk factor for the progression of NAFLD [3]. VAT surrounds the abdominal organs in the abdominal cavity. Its volume increase is a risk factor for abdominal obesity, insulin resistance and other important cardiovascular diseases and also plays an important role in hepatic steatosis, inflammation and fibrosis. Excessive visceral fat accumulation could release various bioactive adipocytokines [31], which are prone to induce chronic low-grade inflammation and macrophage accumulation in VAT. In addition, infiltrated macrophages could produce pro-inflammatory cytokines and nitric oxide, leading to adipocytokine dysregulation [32]. Decreased adiponectin level is associated with NAFLD [32]. Although we found that BMI, abdominal fat volume and SAT volume were significantly higher in NAFLD patients than in non-NAFLD subjects (*P* < 0.05), they were not independent correlation factors for NAFLD, suggesting that increased VAT volume is more clinically significant for NAFLD.

## Limitations

The current study also has several limitations. First, this study is a retrospective study. NAFLD was diagnosed using CT imaging and liver degeneration was not confirmed by liver biopsy. Meanwhile, although CT is far more convenient than MR, it is not sensitive to low fat concentration and less sensitive than MR-based techniques for diagnosis [33]. Second, there were only 33 NAFLD patients, which accounted for 17.3% of the study population. This ratio is close to the NAFLD prevalence (15%) in the community of China [1]. Third, PET myocardial dynamic imaging after euglycemic-hyperinsulinemic clamp is the “gold standard” for determination of myocardial insulin resistance, but the imaging process is complicated and time-consuming. Previous studies have indicated that fasting myocardial glucose uptake is correlated with insulin resistance [34], but the pathophysiological mechanism underlying the correlation between myocardial glucose uptake and NAFLD and the prognosis of NAFLD still need to be explored prospectively and confirmed by long-term follow-up.

## Conclusions

NAFLD is a multisystem disease that affects the liver and a variety of extra-hepatic organs. In this study, we for the first time used PET/CT to observe the differences in glucose metabolism levels in different tissues/organs between NAFLD patients and non-NAFLD subjects and elucidated that 1) hepatic steatosis had no significant correlation with hepatic FDG uptake, and patients with ALT ≥50 u/L had increased hepatic fat infiltration but not FDG uptake; 2) NAFLD patients had reduced myocardial glucose metabolism and slightly elevated mediastinal blood pool and spleen SUVmean; 3) reduced myocardial glucose metabolism was one of the independent correlation factors of NAFLD and had a mild linear correlation with hepatic steatosis, and 4) increased serum ALT and VAT volume were also independent correlation factors for NAFLD.

## Data Availability

The datasets used and/or analyzed during the current study are available from the corresponding author on reasonable request.

## Abbreviations

NAFLD: Nonalcoholic fatty liver disease
FDG: ^18^F-fluorodeoxyglucose
PET: Positron emission tomography
CT: Computed tomography
VAT: Visceral adipose tissue
SAT: Subcutaneous adipose tissue
SUVmean: Mean standardized uptake value
NASH: Nonalcoholic steatohepatitis
BMI: Body weight index
ALT: Alanine aminotransferase
AST: Aspartate aminotransferase
TC: Total cholesterol
TG: Triglyceride
HDL-C: High-density lipoprotein cholesterol
LDL-C: Low-density lipoprotein cholesterol
TSH: Thyroid stimulating hormone
HOMA-IR: Homeostasis model assessment of insulin resistance
